# The Methodist-Episcopal Hospital Reports, Brooklyn, N. Y.

**Published:** 1899-10

**Authors:** 


The Methodist-Episcopal Hospital Reports, Brooklyn, N. Y. Volume I., 1887-1897. Edited by Lewis Stephen Pilcher, M. D., and Glentworth Reeve Butler, M. D. New York: Published by the Hospital. 1898.

The board of managers of the Methodist Episcopal Hospital, of Brooklyn, voted on January 27, 1887, to make a contract for the publication of 1,000 copies of a medical and surgical report, to be prepared in view of the tenth anniversary of the hospital and called for one physician and one surgeon from the staff to edit the same. The staff wisely selected Drs. Pilcher and Butler and the wisdom of their choice is clearly shown in the handsome volume prepared. The editors had the hearty coöperation of the attending staff and of others who have been former members of the house staff. Of course, only a small part of the material accumulated has been utilised in this volume. The affections studied have been chosen because of the degree with which they illustrate the practical application of the teachings and resources of the medical and surgical knowledge of the period, and present clearly the nature and scope of the work which the hospital is doing. Many subjects have been necessarily omitted and the authors hold out the hope that they will find full presentations in subsequent volumes, a hope that all those who have the good fortune to secure a copy of this report, will wish to see materialised.

As this is the first publication of the kind emanating from this hospital, the medical and surgical essays are preceded by an historical narrative and a description of the building, and of the administrative organisation, furnished by the administrative officers of the hospital.

Naturally, the frontispiece is a beautiful engraving of George I. Seney, to whose generosity and benevolence the hospital is due. It is a pity that more men like George I. Seney are not among us, especially at this end of the state. The buildings and grounds are well described and the different groups of buildings are shown from the different streets, enabling one to gain a good idea of the noble institution which is doing such excellent work in relieving "Jew and Gentile, protestant and catholic, heathen and infidel, all on the same terms," perpetuating the wish of its noble founder and benefactor.

The essays include the following: Surgical reports; clinical studies of the surgical diseases of the female generative organs, L. Stephen Pilcher. II. Observations upon the injuries of the cranium and of the spine, by George Ryerson Fowler. III. Clinical studies in appendicitis, by George Ryerson Fowler. IV. The cases of fracture of bones, by James P. Warbasse. V. The major amputations, by Thomas Bray Spence. VII. The skin graftings, by Henry Beeckman Delatour; The cases of tetanus, by Charles Henry Goodrich. VIII. The anesthetisations, by Henry Goodwin Webster. IX. Traumatic ruptures of the abdominal viscera, by William Nathan Belcher. X. Surgical operating-room arrangements and methods, by Lewis Stephen Pilcher.

Medical reports: I. The case of typhoid fever, by Alexander Ross Matheson. II. A case of Gilles de la Tourette's disease, by

Alexander Ross Matheson. III. Notes and clinical reports on some pleural and pulmonary conditions, by Glentworth Reeve Butler. IV. The cases of aneurism occurring in the medical service, by William Nathan Belcher. V. The cases of acute (non-alcoholic) poisoning, by Ralph Melville Mead. VI. The cases of uterine fever and heat prostration, by Frank Whitfield Shaw. VII. The cases of diseases of the lungs, bronchi and pleura, by Frank Whitfield Shaw. VIII. The cases of diseases of the liver, by Arthur Henry Bogart. IX. The cases of gastro-intestinal disease, by Charles Richard Butler. X. The cases of nephritis, by Henry Goodwin Webster. XI. The cases of rheumatism, by Raymond Clark. XII. The cases of pregnancy, by Henry Pelouze de Forest. Pediatric reports: I. Empyema of the thorax in children by John Bion Bogart.

Many of the papers are handsomely illustrated, especially Dr. Pilcher's on the surgical diseases of the female generative organs; Dr. Fowler's on clinical studies in appendicitis; Dr. Warbasse on fractures and Dr. Belcher's on traumatic ruptures of the abdominal viscera. It would be impossible to speak of the excellency of all the papers; they are all interesting and scientifically presented and must be of some worth in order to have passed through such able editorial censorship of Drs. Pilcher and Butler. W. C. K.

